# Supplementary material for: The interactome of intact mitochondria by cross-linking mass spectrometry provides evidence for coexisting respiratory supercomplexes
Source: Mol Cell Proteomics. 2017 Dec 8;17(2):216–32. doi: 10.1074/mcp.RA117.000470 (PMC5795388; doi:10.1074/mcp.RA117.000470)
Supplement: Supplemental Data [file supp_RA117.000470_133922_0_supp_23978_fzffwf.zip › spectra_annotation/mito_spectra_annotation/487-1-4-1-10-1.pdf]

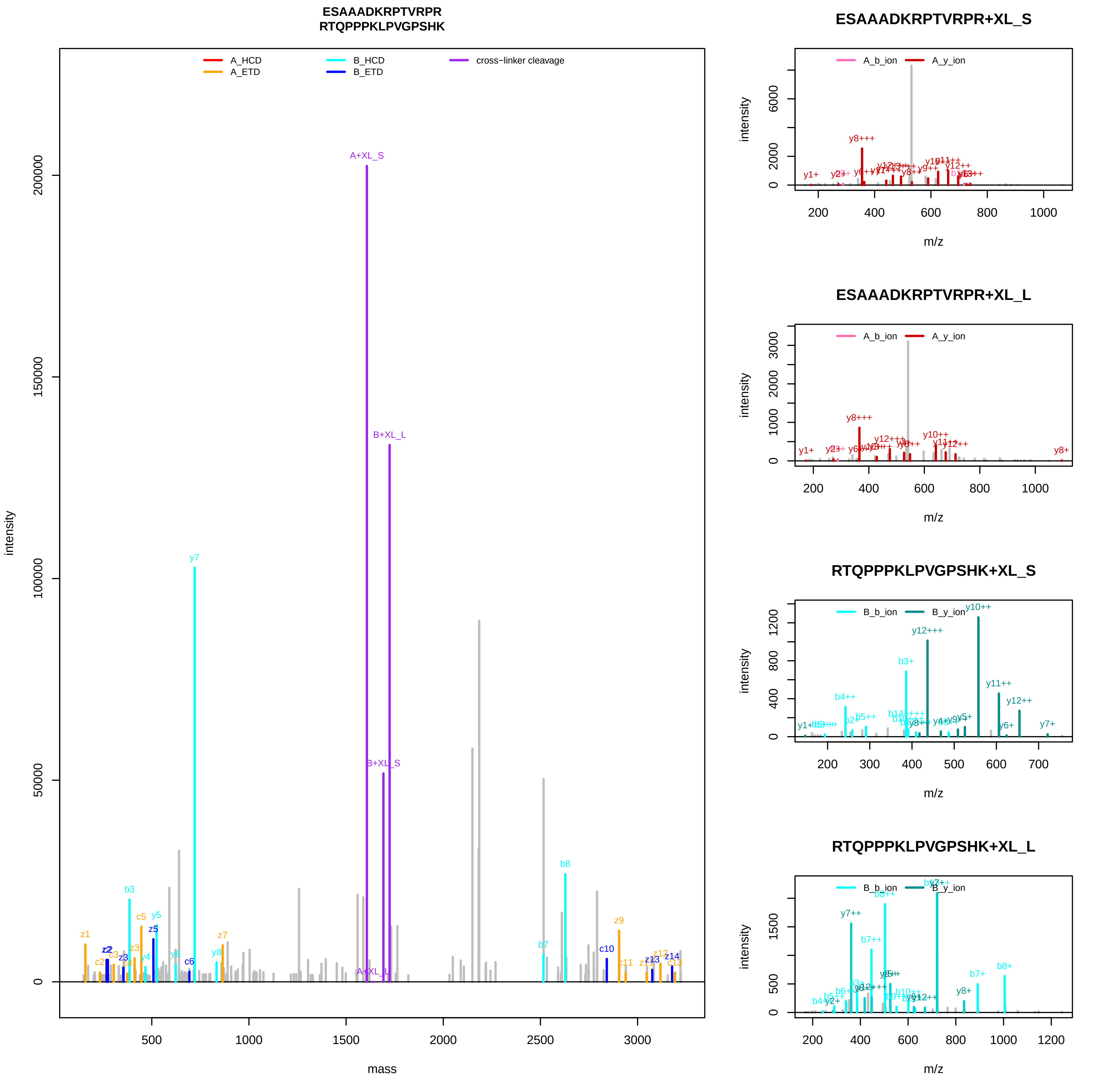

ESAAADKRPTVRPR
RTQPPPKLPVGPSHK
A_HCD
A_ETD
B_HCD
B_ETD
cross−linker cleavage
A+XL_S
B+XL_L
B+XL_S
A+XL_L
y7
b3
b8
b7
c5
y5
z5
z1
z2
z3
c2
c3
c4
z7
y8
y6
c6
y4
c10
z9
c11
z13
z14
z12
c13
intensity
mass
0
50000
100000
150000
200000
500
1000
1500
2000
2500
3000
ESAAADKRPTVRPR+XL_S
A_b_ion
A_y_ion
y8+++
y1+
intensity
m/z
0
2000
6000
200
400
600
800
1000
ESAAADKRPTVRPR+XL_L
A_b_ion
A_y_ion
y8+++
y10++
y12++
y8+
y1+
intensity
m/z
0
1000
2000
3000
200
400
600
800
1000
RTQPPPKLPVGPSHK+XL_S
B_b_ion
B_y_ion
y10++
y12+++
b3+
y11++
y12++
b4++
y7+
intensity
m/z
0
400
800
1200
200
300
400
500
600
700
RTQPPPKLPVGPSHK+XL_L
B_b_ion
B_y_ion
y7++
b7++
b7+
b8+
y8+
intensity
m/z
0
500
1500
200
400
600
800
1000
1200
